# Live fast, die soon: cell cycle progression and lifespan in yeast cells

**DOI:** 10.15698/mic2015.03.191

**Published:** 2015-03-02

**Authors:** Javier Jiménez, Samuel Bru, Mariana Ribeiro, Josep Clotet

**Affiliations:** 1 Dept. Ciències Bàsiques, Facultat de Medicina i Ciències de la Salut. Universitat Internacional de Catalunya, Barcelona, Catalonia, Spain.

**Keywords:** cell cycle, lifespan, phosphate, Pho85

## Abstract

Our understanding of lifespan has benefited enormously from the study of a simple model, the yeast *Saccharomyces cerevisiae*. Although a unicellular organism, yeasts undergo many of the processes directly related with aging that to some extent are conserved in mammalian cells. Nutrient-limiting conditions have been involved in lifespan extension, especially in the case of caloric restriction, which also has a direct impact on cell cycle progression. In fact, other environmental stresses (osmotic, oxidative) that interfere with normal cell cycle progression also influence the lifespan of cells, indicating a relationship between lifespan and cell cycle control. In the present review we compile and discuss new findings related to how cell cycle progression is regulated by other nutrients. We centred this review on the analysis of phosphate, also give some attention to nitrogen, and the impact of these nutrients on lifespan.

## INTRODUCTION

The ability to prolong life is perhaps the most pursued of all human desires, at least in the field of medical science. To reach this goal, a great deal of research has been carried out in order to understand how cells age, identify the underlying processes and mechanisms of cell aging, and determine what processes and mechanisms bring about an extended lifespan 1,2.

Lifespan is mainly examined using 2 different concepts: chronological lifespan (CLS) and replicative lifespan (RLS). CLS is the length of time that a non-dividing cell survives. For example, in the context of nutrients as a growth-limiting factor, yeast cells arrest proliferation, mainly in the G_1_ phase of the cell cycle. These cells can survive in this quiescent state for a period of time before favourable nutrient conditions are restored 3. On the other hand, RLS accounts for the number of times a cell can divide (in the presence of nutrients) before the onset of senescence and death by apoptotic-like mechanisms 3 (for a recent review see 4).

Caloric restriction (limited food intake) is recognized in eukaryotic cells from yeast to mammals, as the most reproducible strategy for expanding lifespan, retarding physiological aging and age associated diseases 5. The mechanisms involved, although extensively studied, are not yet clearly known 2,6,7. Nutrient scarcity (not only carbon but also phosphate and nitrogen) also controls cell cycle progression, bringing about a cell arrest in the G_1_ phase of the cell cycle and its entry into a quiescent phase called G_0_ 8,9. Lifespan extension also occurs when cell cycle progression is arrested or slowed down as a consequence of compromised proliferative signalling pathways or conditions of osmotic, oxidative or replicative stress 2.

When all these notions are considered, the evidence that phosphate and nitrogen scarcity have an impact on the regulation of cell cycle progression may lead to the conclusion that they are also involved in lifespan regulation. This hypothesis is reinforced by the latest findings in the field and constitutes in the main idea presented and discussed in the present review: the availability or scarcity of nutrients (other than carbon) can impact the CLS of cells by controlling the cell cycle progression machinery.

The budding yeast *Saccharomyces cerevisiae* has proven to be a good model organism to study the conserved mechanisms that regulate lifespan in eukaryotic cells, providing a great deal of knowledge on this topic 4. In the present review we will compile, discuss and present our view on how the cell cycle impacts lifespan and aging based on the knowledge generated from this yeast model. With this aim in mind, we will briefly introduce the yeast cell cycle and explain how it is controlled.

## THE YEAST CELL CYCLE

As in any other eukaryotic cell, the cell cycle of yeast involves a group of processes ensuring the generation of new "daughter" cells through the duplication and partition of genetic information and cellular components from a "mother" cell. Accuracy is needed for such important processes, and thus the cell cycle is tightly regulated. Cell cycle progression is mainly controlled by a family of proteins called cyclin-dependent kinases (or CDKs) and, as implied by their name, their interaction with proteins that are oscillatorily expressed during the cell cycle called cyclins 10,11,12.

**Figure 1:**
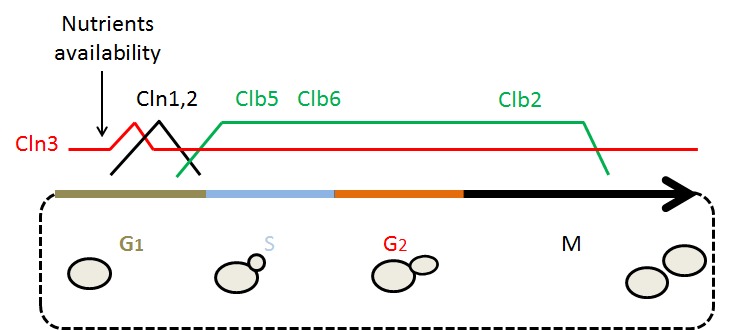
FIGURE 1: The *S.cerevisiae* cell cycle. Schematic representation of the yeast cell cycle phases showing the shape of the cells in each phase. Temporal relationship of the different elements represented in the figure with the cell cycle phases, including relative CDK (cyclin-dependent kinases) activity and the presence of the different cyclins throughout the cell cycle. *The presence of the Cln3 protein is fairly constant throughout the cell cycle but it is only biologically available at the moment indicated.

The cell cycle is composed of 4 different phases: G_1_, S, G_2_ and M. In G_1 _phase, cells prepare for duplication by reaching a threshold of "structures", size or organelles needed to support partition. During the S phase the genetic information is duplicated. In the G_2_ phase the cells get ready for partition. Finally, during the M phase the initial cell is divided into 2 cells (see Fig. 1).

The main CDK in yeast is coded by the gene *CDC28* (CDK1 in mammals and *cdc2^+^* in the fission yeast *Schizosaccharomyces pombe*). Cdc28 can form a complex with 2 families of cyclins known as CLNs and CLBs. The CLB family mainly controls the CDK activity in the S, G_2_ and M phases while the CLN family is circumscribed to the G_1_ phase (for a recent, general review about the yeast cell cycle see 13). We will focus on G_1_ because it is the most relevant stage of the cell cycle with relation to lifespan.

Before starting the treacherous adventure of a new cell cycle round, a G_1_ cell must have available all the "equipment" needed. When a cell reaches the threshold level of confidence, which is measured by the cell in terms of a certain level of chaperones, a CLN called Cln3 is released from chaperone sequestering, interacts with and activates the CDK Cdc28, initiating all the processes that lead cell cycle progression through G_1_ and into S phase 14,15. However, a cell can decide not to follow the G_1_ route after checking the "environment clues" (e.g. Cln3 levels are too low) and enter into a quiescent state called G_0_. G_0_ phase is brought about by the up-regulation of a kinase called Rim15, which is active when environmental conditions are not appropriate for ensuring cell cycle progression 8,9.

The cell cycle machinery is sensitive to many environmental conditions that have a direct effect on cell cycle progression, among them conditions such as osmotic, oxidative or replicative stress. These situations are able to slow down or even arrest cell cycle progression, although by different molecular mechanisms 16,17, to allow adequate time for proper adaptation and successful passage to the next phase.

Nutrient depletion or scarcity can also be considered a stress condition and, as mentioned before, this environmental clue can affect cell cycle progression. From an intuitive point of view, it seems evident that cells would have mechanisms for checking the availability of different nutrients before entering in a new cell cycle round and thus avoid the possibility of cell cycle progression while lacking essential nutrients, which would produce errors in crucial processes such as DNA replication, chromosome segregation, or wall deposition (for an exhaustive review see 18).

**Figure 2:**
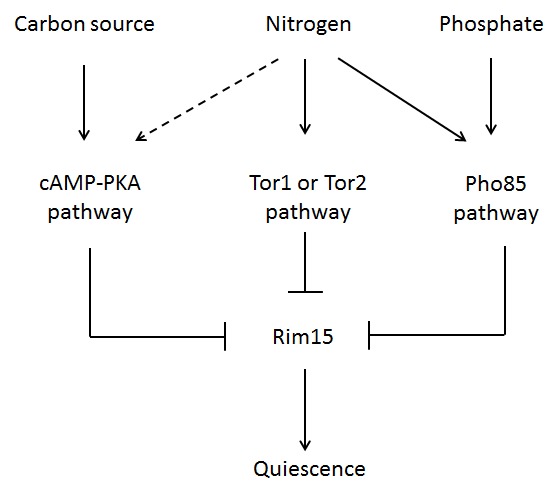
FIGURE 2: Cell cycle control by nutrients in *S. cerevisiae.*

There are some clues about the molecular mechanisms involved in cell cycle exit during nutrient scarcity, and they all involve the up-regulation of the protein kinase Rim15 (Fig. 2). Rim15 can be considered a keystone of many nutrient signalling pathways. Examples include (i) the Ras/PKA pathway when glucose is limiting, (ii) the TOR pathway when nitrogen is scarce or (iii) the Pho pathway when phosphate is the limiting nutrient. The activation of Rim15 leads to G_1_ arrest of the cell cycle, supported by the down-activity of the CDK Cdc28 and/or its associated cyclins, and the subsequent activation of the quiescent state (G_0_) 8,9,19,20. The deletion of the *RIM15* gene induces the transcription of cell cycle-related genes such as the cyclins *CLN1* and *PCL1* (in general, genes with promoter binding domains for Swi4, Swi6 and Mbp1, which are subunits of the MBF and SBF transcription complexes) and other genes involved in phases other than G_1_. In sum, *rim15*∆ cells fail to efficiently curtail cell cycle activity under caloric restriction conditions 19. Rim15 has also been found to play a role in maintaining CLS when yeast cells are fed *ad libitum* and enclosed in beads. Under these unrestricted nutrient conditions, yeast ceases to divide, remains metabolically active, and exhibits no decline in viability over 2 weeks of continuous culture, a condition that produces proliferation arrest in the presence of nutrients. This effect is totally lost in a *rim15*∆ yeast strain 21. Thus, it appears that when the cell cycle is arrested or compromised, for whatever the reason, CLS increases.

Since it is well established that glucose scarcity extends lifespan 4,22,23, the aim of this review will be restricted to the so far not so extensively known roles played by other essential nutrients, namely phosphate and nitrogen (although we will compile mainly the data regarding phosphate), and the recent discoveries about their relationships with cell cycle regulation and its impact on lifespan.

## SENSING AND RESPONDING TO PHOSPHATE: THE Pho PATHWAY

Inorganic phosphate is an essential nutrient for all organisms because it is required for the biosynthesis of nucleotides, phospholipids and some metabolites, making it an important messenger to signal a growth limiting metabolic state and reduced developmental capacities in the cell. Like glucose or nitrogen starvation conditions, the depletion of phosphorous sources forces the yeast cell to enter the quiescent G_0_ state 24.

Intracellular phosphate levels are monitored and homeostatically controlled by the Pho pathway, a pathway that has been progressively elucidated mainly by the group of E.K. O’Shea 25. The Pho pathway is a signal transduction pathway able to sense and respond to the variation of inorganic phosphate and led by the CDK Pho85. Pho85, like other CDKs, must interact with a cyclin to be active. Pho85 binds with 10 different cyclins for its many functions in the biology of *S. cerevisiae* (for recent reviews see 26,27). In phosphate homeostasis the main cyclin is Pho80. Pho85-Pho80 kinase activity is regulated in response to phosphate levels by the CDK inhibitor (CDKI) Pho81 which is always bound to the CDK-cyclin complex, forming a ternary CDK-cyclin-CDKI complex 28. When phosphate becomes limiting, the kinase activity of Pho85-Pho80 is inactivated by Pho81, permitting the dephosphorylation and activation of Pho4 and causing the transcription of genes involved in the survival response to phosphate starvation, such as high affinity phosphate-transporters 28,29,30.

## CELL CYCLE REGULATION BY PHOSPHATE

The group led by Dr. W. Burhans demonstrated that in addition to regulation of the cell cycle machinery and CLS by Rim15 (see above), establishing and maintaining proper arrest in G_1_ is an important cellular response to nutrient deprivation survival. Cells that fail to arrest the cell cycle at G_1_ during nutrient scarcity and proceed through S-phase show DNA replication stress and decreased CLS 31. Cln3 is one of the keystones in driving the cell cycle through G_1_ and into S phase, and the Burhans group showed that *cln3*∆ cells display a lengthened CLS. As could be predicted, the opposite situation can also occur: cells ectopically overproducing Cln3 present a sharply reduced CLS 31. To our knowledge, this is the first report in which an important cell cycle regulator other than Rim15 has been found to also strongly impact CLS. Recently, this result was corroborated by the Deluna group 32, who, using a competitive growth analysis of a yeast gene-deleted collection, found that Cln3 acts as a CLS regulator. Not surprisingly, Cln3 is not the only cyclin involved in CLS regulation. Although the evidence is contradictory and far from being totally understood, the deletion of Cln2 has been shown to reduce CLS 33. The single deletions of the mitotic cyclins Clb1 and Clb2 have been shown to have the same effect 34. It must be stated that all the above-mentioned evidence was obtained from large-scale survey experiments; direct evidence will help to clarify the involvement of these cell cycle players in CLS.

Although several cyclins are involved in CLS regulation, it must be mentioned that in terms of the cell cycle and in concordance with the Burhans group hypothesis, G_1_ is the most relevant phase in cell cycle control and has the biggest influence on CLS. Among the cyclins presented before, Cln3 is the main G_1_ regulator due to its involvement in firing the SBF and MBF promoters, ultimately responsible for leading cells into S-phase. Thus, it seems clear that the downregulation of Cln3 is an essential phenomenon to control both the cell cycle in G_1_ and CLS.

Our group has also participated in identifying Cln3 down-regulation mechanisms, in this case in the context of phosphate deprivation. When phosphate is present, the Pho85/Pho80 complex is active and directly phosphorylates Cln3. When phosphorylated, Cln3 has been proven to be more resistant to proteasome degradation and therefore able to promote a new cell cycle round. When phosphate is limiting, the Pho pathway is inactivated by the presence of the CDK inhibitor Pho81 and, consequently, Cln3 remains unphosphorylated and is quickly degraded by the proteasome 27,35. The absence of Cln3 cannot explain the observed arrest in the absence of phosphate since the *cln3*Δ mutant is still able to enter into the cell cycle in rich media. In this scenario, it is important to mention that Pho85/Pho80 is also able to regulate Rim15 by phosphorylation. Thus, under phosphate-limiting conditions the Pho85-Pho80 complex is inactive and, as a consequence, unphosphorylated Rim15 enters into the nucleus and induces quiescence (G_0_). Putting all together, through a phosphorylation process mediated by the CDK Pho85, phosphate scarcity downregulates Cln3 opposing to G_1_ cell cycle progression and activates Rim15 promoting G_0_ phase and hence moving the balance to promote expanded CLS. Supporting this notion, the presence of a hyperstable allele of Cln3 (*cln3-1*) in phosphate-depleted media increases the number of S phase-arrested cells and decreases cell viability, and cells with a *CLN3* allele that carry aspartic acid substitutions, which mimic Pho85 phosphorylation, also die prematurely 35.

Since Pho85 controls Cln3 stability and cell cycle progression in the absence of phosphate, it is therefore possible to predict that Pho85 is involved in CLS regulation, although the findings to date are controversial. According to the Burtner group 36, the deletion of *PHO85* produces a positive impact on CLS. This result is consistent with the Cln3 phenotype discussed before, but is in stark contrast with the reduced CLS observed by Marek group 33. Both groups carry out large-scale surveys using a gene deletion collection, but the procedures to assess CLS are different: Marek group 33 measured viability after nutrient starvation, while Burtner group 36 followed the outgrowth kinetics of chronologically aged cultures. Obviously, further study is needed to clarify this particular aspect.

**Figure 3:**
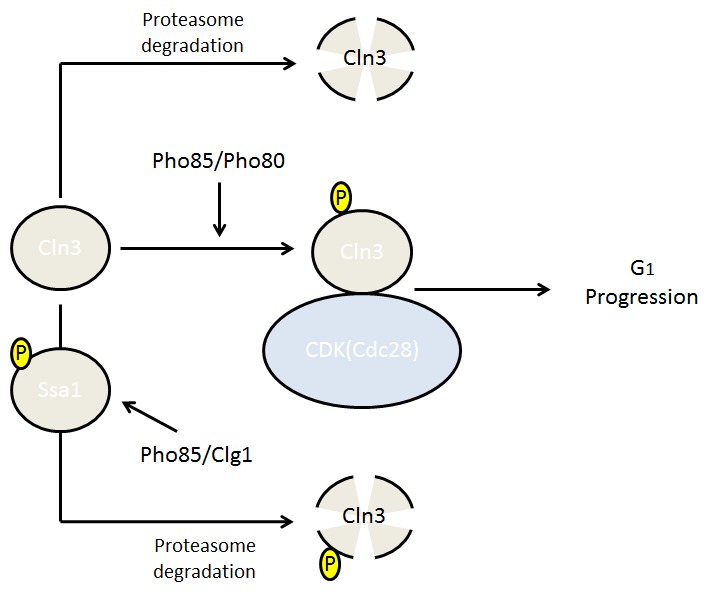
FIGURE 3: Pho85 involvement in cell cycle progression. The cyclin Cln3 is phosphorylated by Pho85-Pho80 as a response to phosphate presence, resulting in increased stability of the cyclin and permitting it to reach the threshold for cell cycle progression through G_1_. Pho85-Clg1 can also phosphorylate Cln3, although at different residues, promoting its degradation as a response to nitrogen scarcity.

Cln3 levels are also regulated by nitrogen 37, making it tempting to speculate about the more general control of G_1_ progression by nutrients and therefore the more general control of CLS - in this case, through nitrogen scarcity. Recently, Dr. Kron group demonstrated that Pho85 is again involved in such regulation, playing an important role in cell cycle regulation during nitrogen scarcity. In this case, Pho85 forms a complex with the cyclin Clg1 and is able to phosphorylate the chaperone protein Ssa1, a chaperone that is essential for Cln3 protection from the degradation machinery 38. Thus, Pho85 appears to control Cln3 stability in different situations of nutrient availability using different molecular mechanisms (Fig. 3), and in consequence, it might be that it also affects CLS.

The picture is not finished yet. In the previously discussed results, Pho85, along with its cyclins Pho80 and Clg1, works actively against Cln3. Thus far, Pho80 has not been shown to be involved in CLS, and, contrary to some predictions, Clg1 deletion was described by Garay group 32 to have a negative impact on CLS. In addition, cells with deleted Pcl1 (another Pho85 cyclin) were shown to present increased CLS 32. This further demonstrates the need to unveil the exact role of Pho85 and its cyclins in controlling CLS.

In summary, the emerging idea regarding phosphate scarcity and lifespan, which is one of the contributions by this review, is that Pho85 activity is almost dispensable when yeast grows in nutrient-rich media, although cells still undergo a longer G_1_ phase, but it is essential in other situations (e.g*.* maintaining an induced G_0_ arrest). Since wild yeast thrive under diverse conditions where the availability of phosphate and nitrogen often varies widely, we postulate that the control of Cln3 by Pho85 should be fundamental to maintaining good levels of CLS in yeast cells under natural conditions.

## CONCLUSION

New research has provided a number of answers to how cell cycle machinery is directly affected by nutrient scarcity, especially in the case of phosphate, and how it may have a direct impact on the lifespan of cells. The idea proposed in this review is that a tight control of cell cycle progression is essential to prolonging lifespan. In this context, a good "brake" (i.e. Rim15) appears to be an important part of cell cycle mechanics when the cell cycle must be arrested, as well as a well-controlled "accelerator" (i.e. Cln3). Less control over the brake or accelerator produces a faster progression through the cell cycle and a reduction in the CLS, mainly due to the difficulty in efficiently arresting the cell cycle when dictated by environment conditions. In other words, cells that "live fast" by passing through the cell cycle due to a lack of control produce a less robust G_1_ arrest, a poorer quiescent state and "die soon" because lifespan is reduced. In situations where cell cycle progression is slowed down by either nutrient scarcity (e.g. Rim15 activation, Cln3 down-regulation) or stress, which is well known to arrest the cell cycle, cells are more proficient in properly arresting the cell cycle at G_1_, entering into the quiescent state and prolonging their CLS.
